# The effect of exogenous melatonin on waterlogging stress in *Clematis*

**DOI:** 10.3389/fpls.2024.1385165

**Published:** 2024-06-18

**Authors:** Kai Chen, Qingdi Hu, Xiaohua Ma, Xule Zhang, Renjuan Qian, Jian Zheng

**Affiliations:** ^1^ College of Landscape Architecture, Zhejiang A & F University, Hangzhou, China; ^2^ Wenzhou Key laboratory of Resource Plant Innovation and Utilization, Institute of Subtropical Crops, Zhejiang Academy of Agricultural Sciences, Wenzhou, China

**Keywords:** *Clematis tientaiensis*, *Clematis lanuginosa*, waterlogging, physiological analysis, transcriptome analysis, transcription factor

## Abstract

*Clematis* is the queen of the vines, being an ornamental plant with high economic value. Waterlogging stress reduces the ornamental value of the plant and limits its application. Melatonin plays an important role in plant resistance to abiotic stresses. In this study, the physiological responses and gene expression levels of two wild species, namely, *Clematis tientaiensis* and *Clematis lanuginosa*, and two horticultural varieties, namely, ‘Sen-No-Kaze’ and ‘Viva Polonia,’ under waterlogging stress were analyzed to determine the effect of melatonin on waterlogging tolerance. The results showed that the waterlogging tolerances of *C. lanuginosa* and ‘Sen-No-Kaze’ were relatively poor, but were significantly improved by concentrations of 100 μmol·L^-1^ and 50 μmol·L^-1^ melatonin. *C. tientaiensis* and ‘Viva Polonia’ had relatively strong tolerance to waterlogging, and this was significantly improved by 200 μmol·L^-1^ melatonin. Under waterlogging stress, the relative conductivity and H_2_O_2_ content of *Clematis* increased significantly; the photosynthetic parameters and chlorophyll contents were significantly decreased; photosynthesis was inhibited; the contents of soluble protein and soluble sugars were decreased. Effective improvement of waterlogging tolerance after exogenous melatonin spraying, the relative conductivity was decreased by 4.05%-27.44%; the H_2_O_2_ content was decreased by 3.84%-23.28%; the chlorophyll content was increased by 35.59%-103.36%; the photosynthetic efficiency was increased by 25.42%-45.86%; the antioxidant enzyme activities of APX, POD, SOD, and CAT were increased by 28.03%-158.61%; the contents of proline, soluble protein, and soluble sugars were enhanced, and cell homeostasis was improved. Transcription sequencing was performed on wild *Clematis* with differences in waterlogging tolerance, and nine transcription factors were selected that were highly correlated with melatonin and that had the potential to improve waterlogging tolerance, among which *LBD4*, and *MYB4* were significantly positively correlated with the antioxidant enzyme system, and *bHLH36*, *DOF36*, and *WRKY4* were significantly negatively correlated. Photosynthetic capacity was positively correlated with *DOF36* and *WRKY4* while being significantly negatively correlated with *MYB4*, *MOF1*, *DOF47*, *REV1* and *ABR1*. Melatonin could enhance the flooding tolerance of *Clematis* by improving photosynthetic efficiency and antioxidant enzyme activity. This study provides an important basis and reference for the application of melatonin in waterlogging-resistant breeding of *Clematis*.

## 1 Introduction

Water is one of the key factors affecting plant growth and development as well as interactions with the environment. In recent years due to global warming, continuous destruction of the environment, and frequent occurrence of extreme weather events, the main causes of waterlogging stress have included extreme precipitation in a short period, poor soil drainage, and heavy soil structure (Fukao et al., 2019). Waterlogging affects crop production on about 10% of global arable land and has become one of the major natural disasters threatening the development of the agricultural industry (Tian et al., 2021; Liu et al., 2023). In southern China, especially in the middle and lower reaches of the Yangtze River, waterlogging disasters often occur due to excessive rainfall, high groundwater levels, and poor soil drainage. This has serious impacts on the external morphology, physiology, and biochemistry of plants (Xia and Chen, 2021; Jiang et al., 2022).

*Clematis* are mostly woody or grassy vines with attractive colors, a rich diversity of flower types, and a long flowering period, attributes that give the plants high ornamental and medicinal value. The plants are called the “Queen of Vines” and are widely used in landscaping and ecological restoration (Hu et al., 2021). *Clematis* is resistant to water and humidity. Long-term exposure to soils with saturated or near-saturated water will cause root rot, leaf wilting, and yellowness, inhibition of plant growth, the decline of ornamental quality, and plant death in severe cases (Rabiza-Świder et al., 2017). More rain in the plum rain season in southeastern coastal areas of China coincides with the flowering period of most *Clematis*, seriously affecting large-scale production and gardening applications (Jiang et al., 2010; Zhu et al., 2024). Therefore, analyzing the waterlogging tolerance of *Clematis* and establishing waterlogging-resistant varieties are key to breeding *Clematis* with high stress tolerance.

In waterlogging, the soil moisture content exceeds the field capacity, and the soil pores are filled with water, which inhibits and reduces the utilization rate of O_2_ by plant cells. As a result, the oxygen supply to the plant root cells is insufficient, and cellular aerobic respiration changes to anaerobic respiration, resulting in the accumulation of respiratory products, such as ethanol, acetaldehyde, and lactic acid, and resulting in the death of root cells. The number of rotten and blackened roots increases (Kurokawa et al., 2018; Hu et al., 2022). The impaired functions of the underground parts, such as water and nutrient absorption, indirectly lead to changes in the functions of the above-ground parts, including stomatal closure, chlorophyll degradation, and cell structure damage, thereby inhibiting gas exchange and photosynthetic efficiency of the leaves and seriously reducing the supply of plant energy materials (Peng et al., 2017; Zeng et al., 2021). The state of high O_2_ content and low CO_2_ content in photosynthetic tissues promotes the consumption of energy resources by enhanced photorespiration of plants, further hindering the accumulation of photosynthetic products and the absorption and transport of nutrients and aggravating the secondary stress responses of the plants (Tong et al., 2021; Zhong et al., 2023).

Under waterlogging stress, the dynamic balance of reactive oxygen species (ROS) is disturbed, resulting in metabolic disorders and electron leakage i*n vivo* and accumulation of ROS, such as superoxide anions (O_2_^−^), hydroxyl radicals (OH^−^), hydrogen peroxide (H_2_O_2_), and singlet oxygen (^1^O_2_) (Singh et al., 2016). ROS accumulation has serious toxic effects on plant cells, and plants maintain ROS metabolic balance by initiating antioxidant and non-enzymatic antioxidant protection mechanisms (Bailey-Serres and Mittler, 2006; Mittler, 2017). Antioxidant enzymes include superoxide dismutase (SOD), peroxidase (POD), catalase (CAT), and ascorbate peroxidase (APX) (Wang et al., 2022a). SOD clears O_2_^−^ to form H_2_O_2_ and O_2_; POD and CAT catalyze H_2_O_2_ to form H_2_O and O_2_, and the three enzymes work synergistically to protect plants from damage (Bela et al., 2015; Zhang et al., 2023a). Non-enzymatic antioxidant protective substances include soluble protein, soluble sugar, and proline, which can synergically enhance the activity of related enzymes or act as substrates for related enzymes to improve the survival ability of plants under abiotic stress (Pardo-Hernández et al., 2020; Wang et al., 2022).

Melatonin (MT), also known as pineal hormone, is an endogenous small-molecule substance that widely exists in animals and plants. It can easily cross the cell membrane and enter the subcellular compartment, and it has a wide range of physiological effects in animals and plants (Sun et al., 2021; Anitha et al., 2022; Eisa et al., 2023). Melatonin is present in more than 20 dicotyledonous and monocotyledonous plants (e.g., *Allium cepa*, *Oryza sativa*, *Solanum lycopersicum*, *Musa nana*, *Malus pumila*, and *Cucumis sativus*) and occurs in roots, stems, leaves, fruits, flowers, and seeds (Nawaz et al., 2016; Wei et al., 2018). Melatonin plays an important role in plant growth and development. Melatonin treatment improved the germination rates of *Limonium bicolor* and *Cucumis melo* L. seeds under salt stress by promoting soluble sugar utilization and the synthesis of new proteins and by increasing amylase and α-amylase activities, respectively (Castañares and Bouzo, 2019; Li et al., 2019).

Melatonin can alleviate chlorophyll degradation, maintain chlorophyll activity, and improve the plant photosynthetic rate in *Arabidopsis thaliana*, *O. sativa*, *Triticum aestivum*, and other plants under normal and abiotic stress conditions (Back, 2021; Talaat, 2021; Yang et al., 2021; Cao et al., 2023). Exogenous melatonin can significantly inhibit the decreases in chlorophyll degradation and photosynthetic rate under salt stress, balance the electron transfer in the donor, acceptor, and reaction center of the PSII system, and thus effectively alleviate the damage from salt stress on the cucumber photosynthetic system (Wang et al., 2016; Jahan et al., 2021). Exogenous application of melatonin can increase the expression of anthocyanin-related genes in tea leaves under arsenic stress, promote the production and accumulation of anthocyanins, and thus alleviate the effects of arsenic stress on the photosystem of tea trees (Li et al., 2021; Langaroudi et al., 2023).

Melatonin is also a powerful free radical scavenger and oxidizing agent that plays a direct role in scavenging free radicals, such as ROS and reactive nitrogen species (RNS), and maintaining the redox balance of plants (Tiwari et al., 2021; Ahmad et al., 2023). Melatonin can induce and enhance the activity of various antioxidant enzymes, thereby improving its efficiency as an antioxidant (Debnath et al., 2019), and its activity is five times that of glutathione (GSH) and 15 times that of mannitol (Bidabadi et al., 2020; NaveenKumar et al., 2020). Melatonin can increase the resistance of wheat to low-temperature stress (Turk et al., 2014; Kumar et al., 2021; Wu et al., 2022). Application of optimum rate of melatonin under high-temperature stress increased the activities of APX and POD by 43.7% and 45.5%, respectively, which had a positive effect on the growth of cherry radish (Jia et al., 2020).

In *Prunus persica* treated with 200 µmol· L^-1^ MT, the activities of superoxide dismutase and peroxidase were increased, while the contents of H_2_O_2_ and ethylene were decreased, indicating that melatonin has an antioxidant effect on waterlogging stress (Gu et al., 2021). Exogenous melatonin enhanced the resistance of pear and *Solanum lycopersicum* to waterlogging as well as to heavy metals and other stresses by increasing the activity of antioxidant enzymes and reducing the contents of hydrogen peroxide and malondialdehyde (Hasan et al., 2019; Liu et al., 2019) Melatonin also reduced low-salt-induced K^+^ efflux in *O. sativa* and reduced the sensitivity of plasma membrane K^+^ permeable channels to hydroxyl radicals (Liu et al., 2020).

There are about 355 species of *Clematis* germplasm resources in the world, and 147 species of wild *Clematis* in China. There are 33 species of wild *Clematis* plants in Zhejiang Province, among which *Clematis tientaiensis* is a unique wild germplasm resource, and *Clematis lanuginosa* is an important breeding parent. A previous study found that *C. lanuginosa* had relatively strong heat resistance, and cultivation showed that *C. tientaiensis* had strong adaptability and resistance to insect pests (Qian et al., 2022). The flower diameter of both species is more than 15 cm, giving the plants high ornamental and breeding value. In this study, two wild germplasm resources with excellent resistance, namely, *C. tientaiensis* and *C. lanuginosa*, as well as the horticultural varieties ‘Sen-No-Kaze’ and ‘Viva Polonia,’ were selected as materials, and exogenous melatonin was used to analyze the physiological responses of the photosynthetic and antioxidant systems of plants under waterlogging stress. The study provides an empirical basis and a reference for exploring the application of breeding new varieties of *Clematis* with high stress resistance.

## 2 Materials and methods

### 2.1 Plant materials and growing conditions

*C. tientaiensis*, *C. lanuginosa*, ‘Sen-No-Kaze’, and ‘Viva Polonia’ were stored in the National *Clematis* germplasm Resource Bank of Zhejiang Institute of Subtropical Crops. Three-year-old healthy *Clematis* plants with consistent growth, and free of pests and diseases were selected and planted in individual pots. The basin diameter was 11 cm, and the basin height was 18 cm. The planting medium contained peat, coconut husk, perlite, and light stone in the ratio 55:15:15:15. The test site was a greenhouse located at E 120°38 '30.99"; and N 28°0' 16.87". The area has a subtropical marine monsoon climate with four distinct seasons and abundant rainfall, with an annual average rainfall of 1700 mm and a frost-free period of 276 days. The average temperature was 31.4 ± 5.0°C. and the relative humidity was 68.0% ± 19.0%.

### 2.2 Waterlogging stress treatment

The double-set basin method was used to carry out flooding stress, and the test design is shown in **Table 1**. The control group (CK) was subjected to normal water management and was watered from 16:00–17:00 every afternoon to maintain the soil water content between 50%–60% until the end of the experiment. For the waterlogging treatment (T0), potted *Clematis* plants were placed in plastic boxes (42 cm long × 31.5 cm wide × 22.5 cm high), keeping the water level 4–5 cm above the soil surface. The medium was replenished regularly, and the water in the plastic box was changed once every seven days for 28 days. Exogenous spraying of melatonin (T1, T2, and T3) was calculated as the first day of stress on the day of waterlogging. The spraying was started at about 17:00 using a fine droplet sprayer and was evenly distributed on the upper and lower surfaces of the leaves, assuming that the surface and back of the leaves were uniformly wet and dripping, and the spraying lasted for seven days. The waterlogging treatment used distilled water instead of exogenous melatonin, and the control group underwent normal water management during spraying. The phenotypes of *Clematis* plants were recorded on the 29th day after the waterlogging treatment. All the healthy and intact leaves were collected and frozen in liquid nitrogen and stored at –80°C for physiological index detection and transcriptome sequencing. Each *Clematis* variety was set up with five potted plants in each treatment for technical replication, with a total of 100 plants in four varieties. The experiment was repeated three times.

**Table 1:** Experimental design of effects of different concentrations of exogenous melatonin on *Clematis* under waterlogging stress.

*Clematis* species	CK	Waterlogging			
		T0	T1	T2	T3
*C. lanuginosa*	CK	Distilled water	MT50μmol·L^-1^	MT 100μmol·L^-1^	MT 200μmol·L^-1^
*C. tientaiensis*	CK	Distilled water	MT50μmol·L^-1^	MT 100μmol·L^-1^	MT 200μmol·L^-1^
‘Sen-No-Kaze’	CK	Distilled water	MT50μmol·L^-1^	MT 100μmol·L^-1^	MT 200μmol·L^-1^
‘Viva Polonia’	CK	Distilled water	MT50μmol·L^-1^	MT 100μmol·L^-1^	MT 200μmol·L^-1^

### 2.3 Leaf gas exchange parameters and chlorophyll content

Healthy and fully developed leaves were randomly chosen for photosynthetic parameter measurements using an LI-6400 XT portable photosynthesis system (Li-Cor Inc., Lincoln, NE, USA), and equipped with a 6400–18 RGB LED light source. Five leaves were determined for each plant, and five plants were determined for each treatment. The measurements were carried out from 9:00 to 11:00 am. The photosynthetic photon flux density was 1200 μmol m^−2^s^−1^; the CO_2_ concentration was 450 ppm, and the relative humidity was 65%.

The leaves of *Clematis* were washed and dried quickly, and the midvein was cut off. A mixed sample of 0.1 g chopped leaves was weighed and placed in a 10 ml test tube with 8 ml of 80% acetone. Chlorophyll was extracted at 4°C in the dark. The absorbance of the supernatant was measured at 663, 645, and 470 nm using a spectrophotometer (Shimadzu UV-2550, Kyoto, Japan). The total chlorophyll content was calculated according to the Arnon formula (Palta, 1990).

### 2.4 H_2_O_2_ content and relative conductivity

The H_2_O_2_ content was measured according to the method previously described (Ma et al., 2017).

Samples of leaves (0.1 g) were weighed, and 10 ml of distilled water was added. The leaves were soaked for 1 h after vacuuming, and then tested for the conductivity (C1). The tubes with the leaf samples were placed in a boiling water bath at 100°C for 10 min, cooled to room temperature, and tested again for conductivity (C2). The relative conductivity was calculated as C1/C2 (Zhai et al., 2016).

### 2.5 Proline, soluble sugar, and soluble protein contents

The proline content was determined using 0.5 g of fresh leaves and 5 ml of 3% sulfamesalicylic acid homogenate, centrifuged at 4000 × g for 10 min, and the filtrate (2 ml) was mixed with indanhydrin (2 ml) and acetic acid (2 ml) in a test tube. The reaction mixture was incubated in a water bath at 98°C for 30 minutes and then extracted with toluene (5 ml). Toluene with chromophores was aspirated, cooled to room temperature, and the absorbance was measured at 520 nm (Bates et al., 1973).

A soluble sugar test kit (Nanjing Jiancheng Bioengineering Institute, Jiangsu, China) was used for determination. For the extraction, 0.2 g leaf samples were ground with 2 ml of distilled water until evenly mixed, heated in a water bath at 98°C for 10 min, centrifuged at room temperature at 4000 r/min after cooling for 10 min, and the supernatant was collected. The substrate was added with concentrated sulfuric acid, and the reaction was carried out in a boiling water bath at 98°C for 10 min. After cooling to room temperature, the absorbance was measured at 620 nm to calculate the soluble sugar content (Zhao et al., 2019).

Determination of soluble protein content in leaves was performed according to the Coomassie brilliant blue (CBB) G250 method described by Read (Read and Northcote, 1981). After adding 5 ml of CBB G-250 reagent to 0.1 ml of enzyme solution, the soluble protein content was determined at 595 nm colorimetric wavelength after standing for 2 min.

### 2.6 Antioxidant enzyme activity

To determine antioxidant enzyme activity, 0.3 g of fresh leaf tissue was homogenized with 8 ml of phosphate buffer (0.1 M, pH 6.8). The mixed liquid sample was centrifuged at 12,000 × g at 4°C for 20 minutes. The obtained supernatant was used to determine the activities of SOD, POD, CAT, and APX. The activity of SOD was determined according to the instructions of a total superoxide dismutase (T-SOD) detection kit (Nanjing Jiancheng Bioengineering Institute, Jiangsu, China). One unit of SOD activity is defined as the amount of enzyme required to inhibit 50% of the reduction rate monitored at 550 nm. The activity of APX was determined using the APX detection kit (Nanjing Jiancheng Bioengineering Institute, Jiangsu, China). APX activity was determined based on the absorbance variation of 290 nm (A10s and A130s). CAT and POD activities were determined by CAT assay kit and POD assay kit (Nanjing Jiancheng Bioengineering Institute, Jiangsu, China), respectively.

### 2.7 Transcriptome analysis

RNA was extracted from *C. tientaiensis* and *C. lanuginosa* with Trizol reagent (Takara, Beijing, China). Total RNA was extracted from tissue samples, and the concentration and purity of the extracted RNA were detected by using a Nanodrop 2000 (Thermo Scientific, Waltham, MA, USA). RNA integrity was detected by agarose gel electrophoresis, and RIN values were determined using an Agilent 5300 analyzer (Agilent Technologies, Palo Alto, CA, USA). After the fragmentation buffer was added, the mRNA was randomly fragmented, and the fragments of about 300 bp were separated by magnetic bead screening. A cDNA library was synthesized by reverse transcription. Double-ended sequencing was performed by second-generation sequencing (Illumina Novaseq 6000, San Diego, CA, USA), and sequencing was performed by Majorbio Biomedical Technology Co., Ltd. (Shanghai, China).

Trinity software (r20140717) was used to splice clean reads to obtain the transcripts for subsequent analysis. NR, Swiss-Prot, eggnog, and KEGG databases (www.kegg.jp/kegg/) were used to annotate all of the unigenes (E value < 1.0 e^−5^). Gene expression was analyzed by FPKM (Fragments Per Kilobase of exon model per Million mapped reads). The criteria for screening DEGs were a *P*-value ≤ 0.05, a false discovery rate (FDR) < 0.001, and a fold difference ≥ 2 or a fold difference ≤ 0.5 (Winfield et al., 2010; MacWilliams et al., 2023).

Using the FPKM values of differentially expressed genes obtained by RNA-seq sequencing, filtering unannotated differentially expressed genes and five genes with the sum of treated expression amounts less than 500, combined with the determined relevant physiological indicators, weighted correlation network analysis (WGCNA) was performed to construct a correlation matrix between phenotypes and gene modules (Azam et al., 2023; Wang et al., 2023).

Total RNA was extracted from leaves, and cDNA was synthesized with a reverse transcription kit (Thermo Scientific, Waltham, MA, USA). RT-PCR was performed using an ABI PRISM 7500 real-time PCR system (Applied Biosystems, Foster City, CA, USA) and AceQ qPCR SYBR Green Master Mix (Vazyme, Nanjing, Jiangsu Province, China). The RT-PCR reaction system was as follows: 5 min at 95°C, 40 cycles at 95°C for 15 s, and 30 cycles at 60°C. Each sample was repeated three times, and the reference gene was GAPDH. The specificity of each pair of primers was verified by a melting curve analysis, and the gene expression levels were calculated by the 2^−△△Ct^ method.

### 2.8 Statistical analysis

SPSS22.0 statistical software was used to conduct two-way ANOVA for all of the data, and all of the charts were produced by GraphPad Prism 9.5.1 and SigmaPlot 12.5 software. Different letters on the histograms between different treatments indicate a significant difference at *P* ≤ 0.05.

## 3 Results

### 3.1 Exogenous melatonin improved the growth of *Clematis* after waterlogging stress

**Figure 1:**
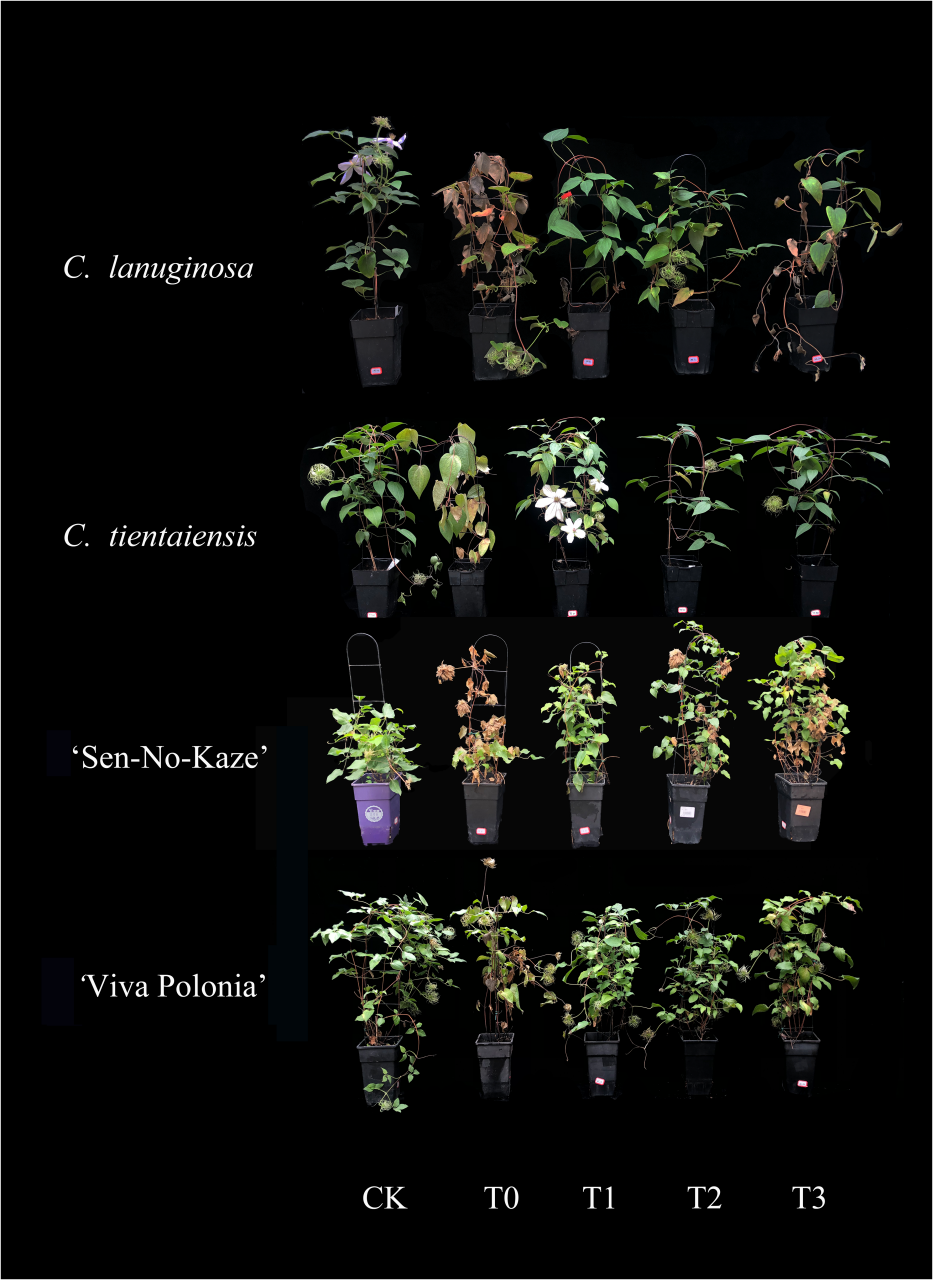
Plant growth of *Clematis* under waterlogging stress treated with different concentrations of exogenous melatonin.

*C. tientaiensis*, *C. lanuginosa*, ‘Sen-No-Kaze’, and ‘Viva Polonia’ showed different degrees of leaf wilting, drooping, yellowing, and drying under waterlogging stress. *C. lanuginosa* and ‘Sen-No-Kaze’ were the most sensitive; most of the leaves withered, drooped, and rotted, while *C. tientaiensis* and *‘*Viva Polonia’ were relatively better, where only a few leaves turned brown and withered. More than 60% of the leaves remained green. The growth of the four species of *Clematis* was improved after exogenous melatonin spraying. Under waterlogging stress, *C. lanuginosa* grew best under T2, while some leaves wilted and yellowed under T3. *C. tientaiensis* and *‘*Viva Polonia’ showed good growth under melatonin treatments, and T3 produced the best growth. *‘*Sen-No-Kaze’ grew best under T1, and wilting and yellowing occurred with T3 (**Figure 1**).

**Figure 2:**
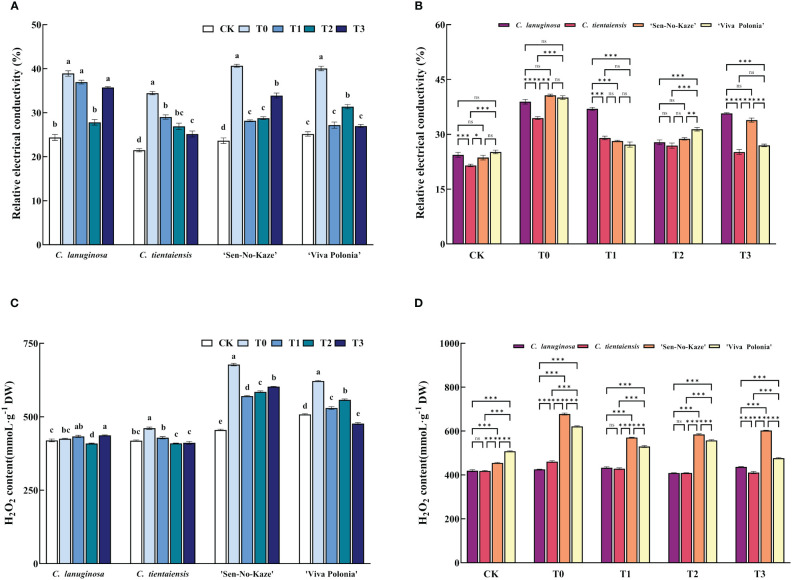
The effects of waterlogging stress and different concentrations of exogenous melatonin on REC and H_2_O_2_ contents of *Clematis*. **(A, B)** Relative electrical conductivity; **(C, D)** H_2_O_2_ contents. Error bars indicate SE (n = 5 plants). Different letters indicate significant differences based on two-way ANOVA followed by Tukey’s multiple comparison test (*P* ≤ 0.05). *means a significant difference at the *P* ≤ 0.05 level; **means a significant difference at the *P* ≤ 0.01 level, ***means a significant difference at the *P* ≤ 0.001 level, and ns means no significant difference at the *P* ≤ 0.05 level.

Waterlogging stress significantly increased the relative electrical conductivity (REC) of *Clematis* leaves, and after exogenous melatonin spraying, the REC of the leaves of the four *Clematis* leaves decreased significantly, among which the relative electrical conductivity of *C. lanuginosa* under T2 decreased significantly by 28.51%, and that of *C. tientaiensis* under T3 decreased significantly by 26.99%. ‘Sen-No-Kaze’ decreased significantly under T1 by 30.75%. ‘Viva Polonia’ had a significant 32.66% decrease under T3 (**Figure 2A**). After waterlogging stress, the REC of *C. tientaiensis* was significantly lower than that of the other three *Clematis*, and the REC of *C. lanuginosa* under the T1 treatment was the highest, while the REC of *C. tientaiensis* and *‘*Viva Polonia’ under the T3 treatment was significantly lower than those of *C. lanuginosa* and ‘Sen-No-Kaze’(**Figure 2B**).

Waterlogging stress also increased the H_2_O_2_ content of *Clematis*. After the addition of exogenous melatonin, the H_2_O_2_ content of *C. lanuginosa* was significantly decreased under T2 (3.84%), and those of *C. tientaiensis* and *‘*Viva Polonia’ were significantly decreased under T3 (10.82% and 23.28%). ‘Sen-No-Kaze’ decreased significantly in T1 by 15.83% (**Figure 2C**). For the different *Clematis* species, the H_2_O_2_ contents of *‘*Viva Polonia’ and *‘*Sen-No-Kaze’ were significantly higher than those of *C. lanuginosa* and *C. tientaiensis*. With the increase of the melatonin concentration, the H_2_O_2_ content in *‘*Viva Polonia’ was similar to those in *C. lanuginosa* and *C. tientaiensis*, but the H_2_O_2_ content in *‘*Sen-No-Kaze’ was significantly higher than that in the other species of *Clematis* (**Figure 2D**).

### 3.2 Exogenous melatonin enhances photosynthesis of *Clematis* under waterlogging stress

**Figure 3:**
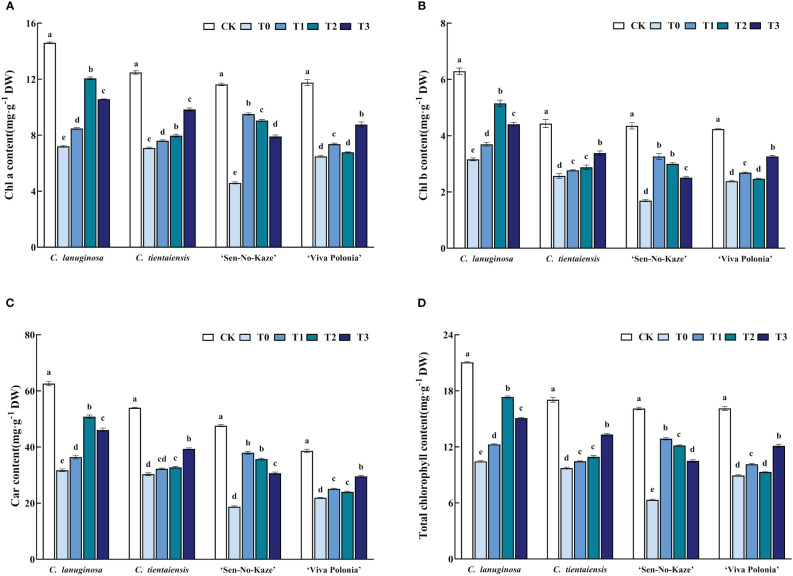
Effects of waterlogging stress and different concentrations of exogenous melatonin on the pigment of *Clematis* leaves. **(A)** Chla content; **(B)** Chlb content; **(C)** Car content; **(D)** Total chlorophyll content. Error bars indicate SE (n = 5 plants). Different letters indicate significant differences based on two-way ANOVA followed by Tukey’s multiple comparison test (*P* ≤ 0.05).

Under waterlogging stress, the contents of Chla, Chlb, Car, and total chlorophyll in the leaves of the four *Clematis* were significantly decreased, and the pigment content was increased by melatonin, but there were differences in the responses to different concentrations. Under the T2 treatment, the contents of Chla, Chlb, Car, and total chlorophyll increased the most compared with waterlogging stress in *C. lanuginosa* and were significantly increased, by 67.32%, 62.96%, 59.99%, and 66.00%, respectively. Under the T3 treatment, the contents of Chla, Chlb, Car, and total Chlorophyll increased the most in *C. tientaiensis*, by 38.14%, 31.56%, 29.88%, and 36.93%, respectively, and in *‘*Viva Polonia’, the respective values were 35.10%, 36.95%, 34.97%, and 35.59%. Under the T1 treatment, the contents of Chla, Chlb, Car, and total Chlorophyll increased the most in ‘Sen-No-Kaze’, by 83.58%, 93.05%, 102.65%, and 103.36%, respectively (**Figure 3**).

**Figure 4:**
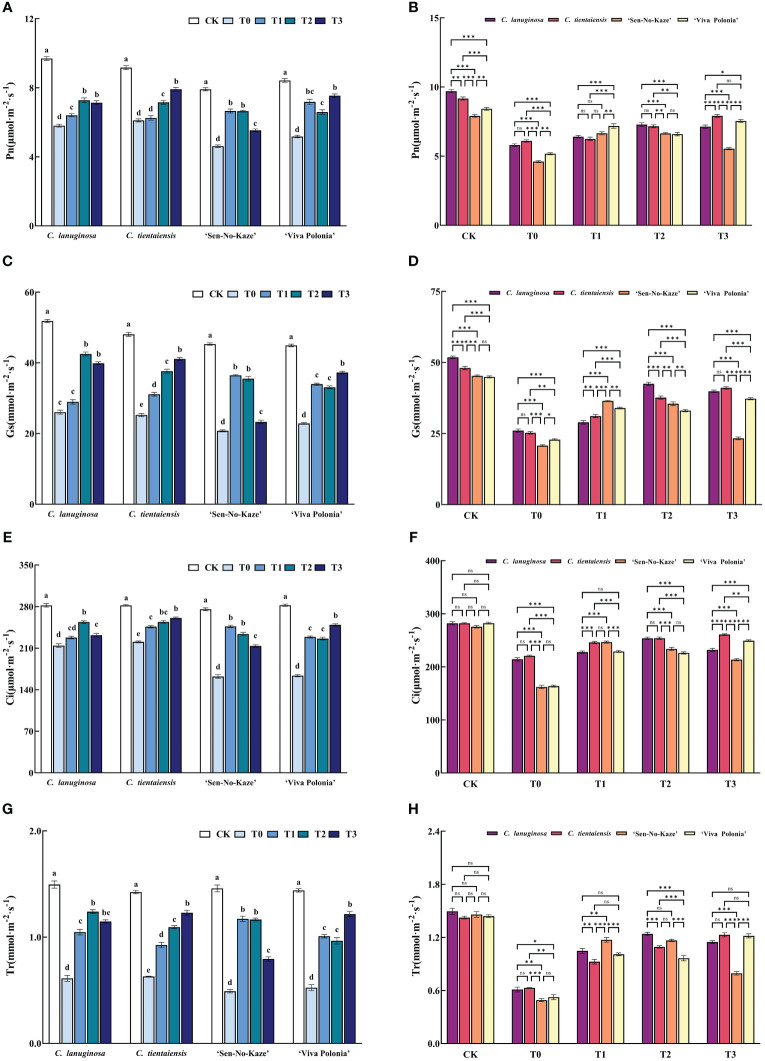
Effects of waterlogging stress and different concentrations of exogenous melatonin on photosynthetic parameters of *Clematis*. **(A, B)** Net photosynthetic rate (Pn); **(C, D)** Stomatal conductance (Gs); **(E, F)** Intercellular CO_2_ concentration (Ci); **(G, H)** Transpiration rate (TR). Error bars indicate SE (n = 5 plants). Different letters indicate significant differences based on two-way ANOVA followed by Tukey’s multiple comparison test (*P* ≤0.05). *means a significant difference at the *P* ≤ 0.05 level. **means a significant difference at the *P* ≤ 0.01 level. ***means a significant difference at the *P* ≤ 0.001 level, and ns means no significant difference at the *P* ≤ 0.05 level.

Waterlogging stress and different concentrations of exogenous melatonin significantly decreased the net photosynthetic rate (Pn), stomatal conductance (Gs), intercellular CO_2_ concentration (Ci), and transpiration rate (Tr) of *Clematis*, and the addition of exogenous melatonin significantly increased photosynthetic gas exchange parameters. Compared to T0, the values of Pn, Gs, Ci, and Tr were increased by 25.42%, 63.22%, 18.47%, and 102.80%, respectively, in the T1 treatment of *C. lanuginosa.* Under T3 conditions, the values of Pn, Gs, Ci, and Tr were significantly increased by 29.57%, 45.86%, 62.77%, and 62.93%, respectively, in *C. tientaiensis*, and by 18.19%, 52.38%, 95.36%, and 132.44% in ‘Viva Polonia’, respectively. In ‘Sen-No-Kaze’, Pn, Gs, Ci, and Tr were increased by 44.25%, 75.64%, 52.11%, and 139.01% under T1 conditions (**Figure 4**).

### 3.3 Exogenous melatonin increased antioxidant enzyme activity of *Clematis* under waterlogging stress

**Figure 5:**
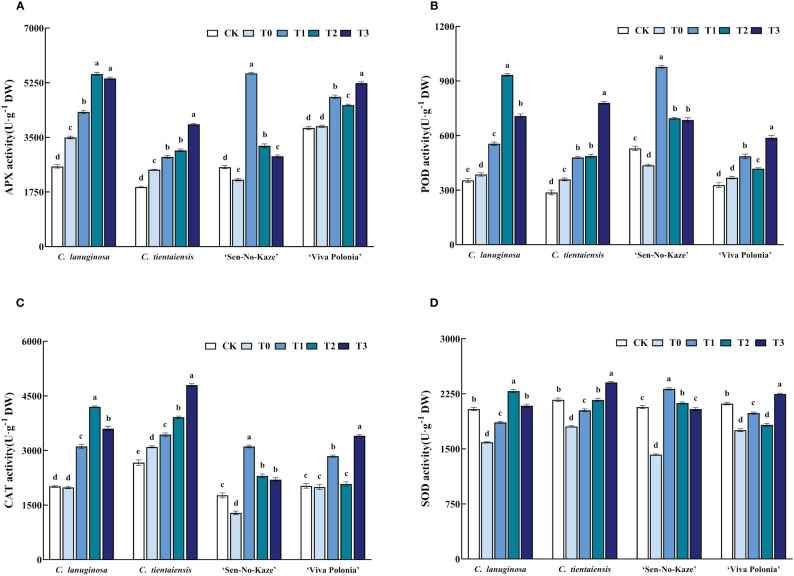
Effects of waterlogging stress and different concentrations of exogenous melatonin on antioxidant enzyme activity of *Clematis*. **(A)** APX activity; **(B)** POD activity; **(C)** CAT activity; **(D)** SOD activity. Error bars indicate SE (n = 5 plants). Different letters indicate significant differences based on two-way ANOVA followed by Tukey’s multiple comparison test (*P* ≤ 0.05).

Waterlogging stress increased the activities of APX, POD, SOD, and CAT in *Clematis* plants, and exogenous melatonin increased the antioxidant activity of *Clematis* under waterlogging stress. Under the T2 treatment of *C. lanuginosa*, the activity of antioxidant enzymes increased the most; APX increased by 58.22%; POD increased by 141.91%; SOD increased by 43.90%, and CAT increased by 111.45%. *C. tientaiensis* and *‘*Viva Polonia’ had the highest antioxidant enzyme activities under T3; APX increased by 59.14% and 35.89%, POD increased by 117.23% and 59.85%; SOD increased by 33.21% and 28.03%, and CAT increased by 54.65% and 70.64%, respectively. Under the T1 treatment, the activities of antioxidant enzymes in *‘*Sen-No-Kaze’ increased the most, with APX being increased by 158.61%, POD increased by 123.91%, SOD increased by 62.99% and CAT increased by 141.72% (**Figure 5**).

### 3.4 Exogenous melatonin increased the content of osmoregulatory substances in *Clematis* under waterlogging stress

**Figure 6:**
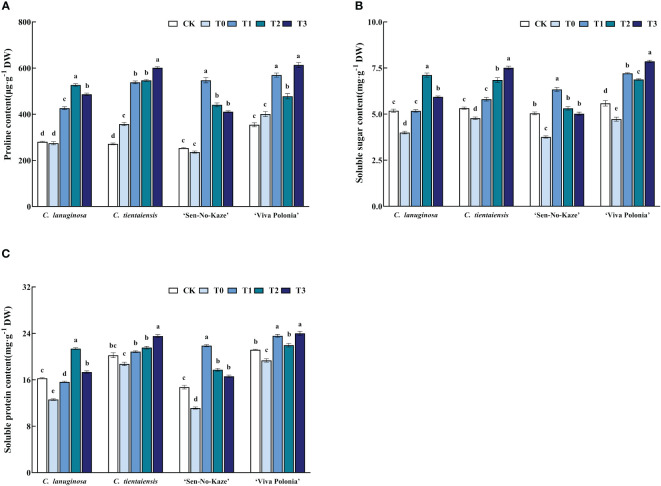
Effects of waterlogging stress and different concentrations of exogenous melatonin on the content of osmoregulatory substances in *Clematis*. **(A)** Proline content; **(B)** Soluble sugar content; **(C)** Soluble protein content. Values are the means ± standard error (n = 5 plants). Different letters indicate significant differences based on two-way ANOVA followed by Tukey’s multiple comparison test (*P* ≤ 0.05).

Waterlogging stress significantly increased the proline content in *Clematis*, and after exogenous melatonin spraying, the proline content was also significantly increased compared to waterlogging stress. For the optimal concentration of melatonin, *C. lanuginosa* significantly increased the proline content by 92.01%, and *C. tientaiensis* significantly increased proline by 68.35%. *‘*Sen-No-Kaze’ showed a significant increase of 131.86%, and *‘*Viva Polonia’ showed a significant increase of 52.98% (**Figure 6A**).

Soluble sugar content was significantly increased after melatonin spraying. In *C. lanuginosa*, soluble sugar was significantly increased by 77.97%; in *C. tientaiensis* by 57.21%, in ‘Sen-No-Kaze’ by 68.64%, and in ‘Viva Polonia’ by 66.35% (**Figure 6B**). Waterlogging stress and exogenous melatonin spraying also increased the soluble protein content in *Clematis* leaves, by 69.83%, 25.67%, 96.67%, and 24.14%, respectively (**Figure 6C**).

### 3.5 Transcriptome analysis of exogenous melatonin supplementation under waterlogging stress

**Figure 7:**
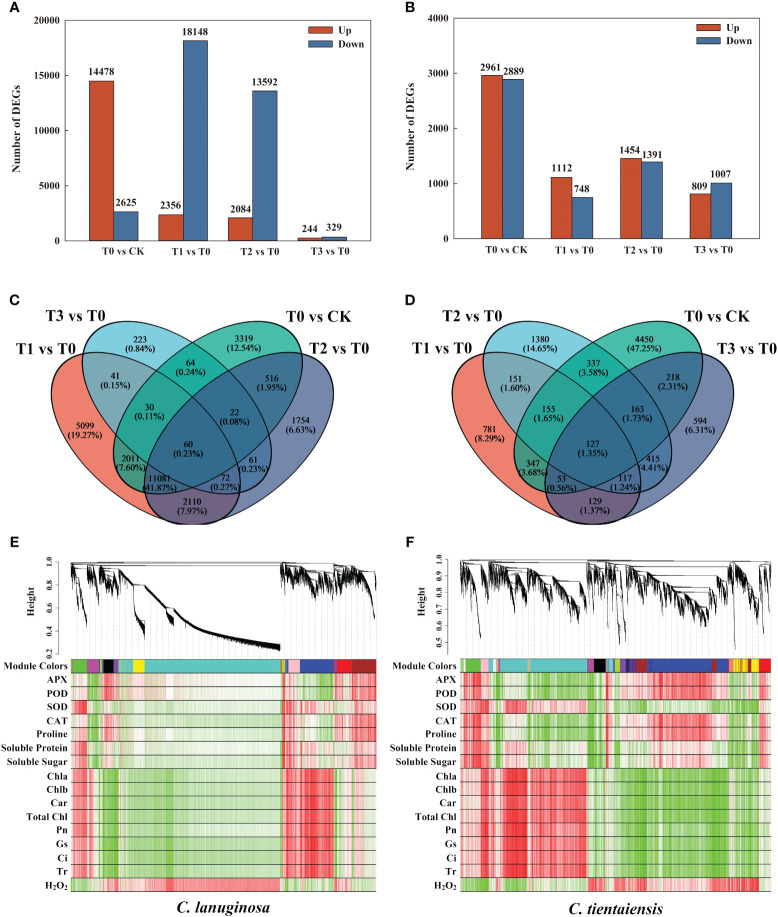
Transcriptome differential genes and association analysis under waterlogging stress and exogenous melatonin treatments. **(A)** The number of DEGs for *C. lanuginosa*; **(B)** The number of DEGs for *C. tientaiensis*; **(C)** Venn diagrams for DEGs in the four comparison groups of *C. lanuginosa*; **(D)** Venn diagrams for DEGs in the four comparison groups of *C. tientaiensis*; **(E)** WGCNA analysis of *C. lanuginosa*; **(F)** WGCNA analysis of *C. tientaiensis*.

*C. lanuginosa* and *C. tientaiensis* were the original breeding parents of *Clematis*, and the two had large differences in waterlogging tolerance. Therefore, transcriptome analysis was conducted for *C. lanuginosa* and *C. tientaiensis*. A total of 199,498 transcriptomes were assembled, comprising 239,096,966 bp of transcript data, of which 129,833 Unigenes were assembled from 153,185,608 bp (**Supplementary Tables S1, S2**). In *C. lanuginosa*, 478 Unigenes were up-regulated, and 2625 were down-regulated under waterlogging stress. For the melatonin treatments T1, T2, and T3, 2356, 2084, and 244 Unigenes were up-regulated, and 18148, 13592, and 329 Unigenes were down-regulated, respectively (**Figure 7A**). In *C. tientaiensis*, 2961 Unigenes were up-regulated, and 2889 were down-regulated under waterlogging stress; 1112, 1454, and 809 Unigenes were up-regulated, and 748, 1391, and 1007 Unigenes were down-regulated under the T1, T2, and T3 treatments, respectively (**Figure 7B**).

A total of 60 genes were differentially expressed (DEGs) in all of the treatments of *C. lanuginosa*; 132 genes (0.50%) were differentially expressed in the melatonin treatment, and 5099 DEGs (19.27%) were specifically expressed in T1 (**Figure 7C**). In *C. tientaiensis*, a total of 127 genes (1.35%) were differentially expressed in all of the treatments; there were 244 DEGs (2.59%) in the melatonin treatment, and 781 DEGs (8.29%) in the T1 treatment (**Figure 7D**). GO analysis revealed that the up-regulated DEGs were annotated in the processes of heterocyclic compound binding, organic cyclic compound binding, and ion binding after the addition of melatonin to *C. lanuginosa* and *C. tientaiensis* under waterlogging stress (**Supplementary Figures S4A, B**). The down-regulated DEGs were annotated in the processes of organic substance metabolic process, primary metabolic process, and organic cyclic compound binding (**Supplementary Figures S4C, D**).

To determine the correlation between phenotype and gene expression and to identify key waterlogging tolerance candidate genes, a WGCNA analysis showed that the DEGs and physiological indicators in *C. lanuginosa* were clustered into 20 modules, among which 45 grey60 modules composed of DEGs were significantly positively correlated with antioxidant enzyme activity and soluble sugar, and 142 magenta modules composed of DEGs were significantly negatively correlated. Pink modules containing 144 DEGs were positively correlated with leaf pigment content, photosynthetic capacity, and negatively correlated with H_2_O_2_ content (**Figure 7E**). The DEGs and physiological indexes of *C. tientaiensis* were grouped into 12 modules.

Antioxidant enzyme activity and soluble sugar were significantly negatively correlated with pink modules (77 DEGs), while the black modules (82 DEGs) were significantly positively correlated with leaf pigment content, photosynthetic system parameters, and soluble protein, and negatively correlated with H_2_O_2_ (**Figure 7F**).

### 3.6 Screening of transcription factors for improving waterlogging tolerance of *Clematis* by melatonin

The transcription factors in the association analysis were screened. Under waterlogging stress and melatonin treatment, 23 transcription factors belonging to 12 gene families were differentially expressed in *C. lanuginosa*. These included five *MYB*, three *bZIP*, three *C2H2*, two *FAR1*, two *ERF*, two *C2C2*, one *bHLH*, one *WRKY*, one *MADS*, one *HSF*, one *LOB*, and one *B3*. There were 14 transcription factors in *C. tientaiensis*, five *MYB*, two *C2H2*, two *ERF*, one *FAR1*, one *bHLH*, one *WRKY*, one *LOB*, and one *B3*. There were 14 differentially expressed transcription factors in both *C. lanuginosa* and *C. tientaiensis*, and nine were differentially expressed in *C. lanuginosa* (**Supplementary Table S3**).

**Figure 8:**
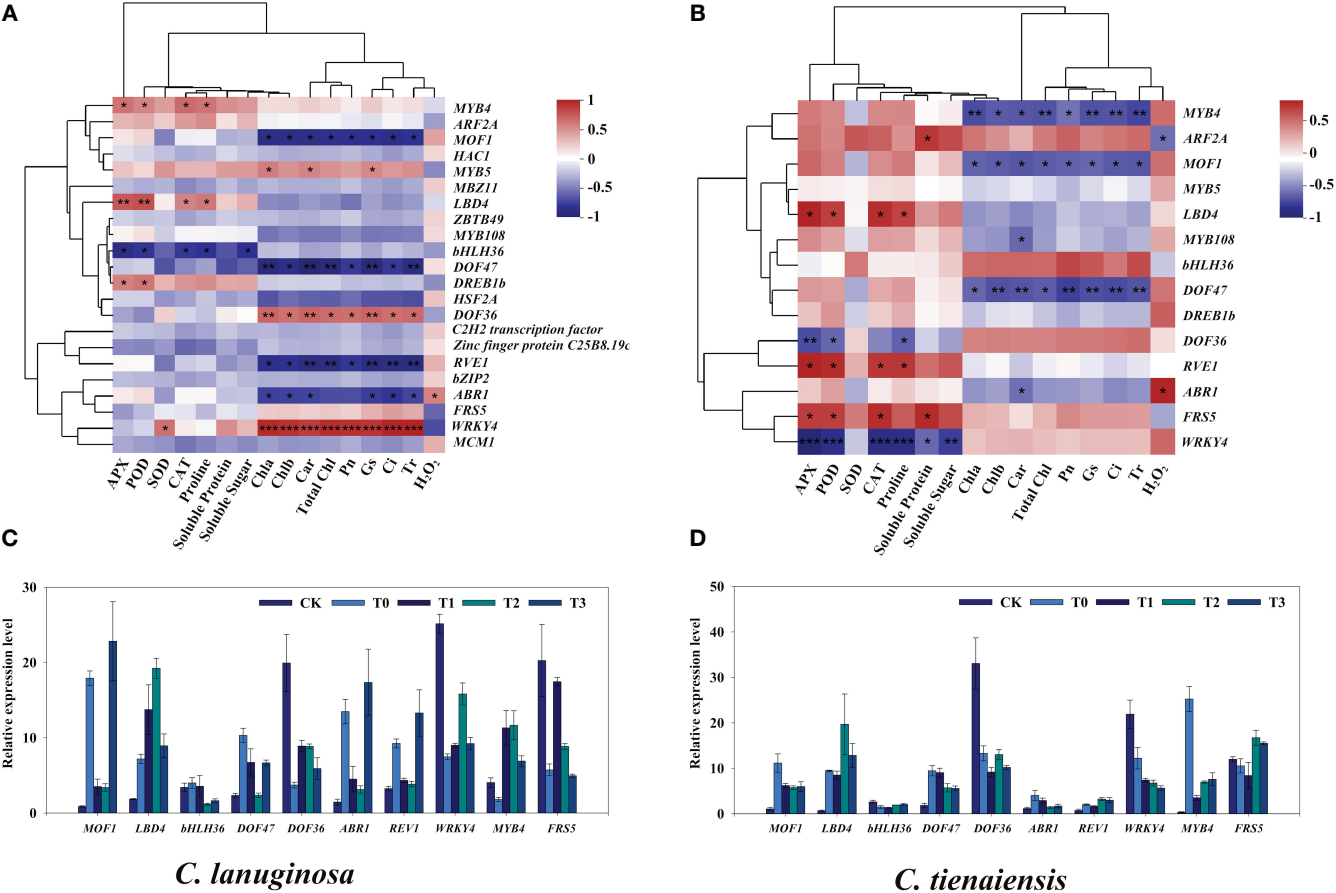
Screening of transcription factors related to waterlogging tolerance of *Clematis*. **(A)** Correlation analysis of different expressed transcription factors and physiological indexes of *C. lanuginosa*; **(B)** Correlation analysis of different expressed transcription factors and physiological indexes of *C. tientaiensis*; **(C)** Transcription factor expression in *C. lanuginosa*; **(D)** Transcription factor expression in *C. tientaiensis*. *means a significant difference at the *P* ≤ 0.05 level. **means a significant difference at the *P* ≤ 0.01 level. ***means a significant difference at the *P* ≤ 0.001 level.

Transcription factors in significantly correlated modules were further identified. In *C. lanuginosa*, *LBD4*, *MYB4*, and *DREB1b* were significantly positively correlated with antioxidant enzyme activity; *bHLH36* was significantly negatively correlated with antioxidant enzyme activity and soluble sugar content, and the transcription factors that were significantly positively correlated with photosynthetic system parameters were *MYB5*, *DOF36* and *WRKY4*. The negative correlations involved *MOF1*, *DOF47*, *REV1* and *ABR1* (**Figures 8A, B**). In *C. tientaiensis*, *LBD4*, *REV1*, *FRS5*, antioxidant oxidase activity, and soluble sugar content were significantly positively correlated; *DOF36 and WRKY4* was significantly negatively correlated, and photosynthetic system parameters were significantly negatively correlated with *MYB4*, *MOF1*, and *DOF47* (**Figures 8C, D**). To further verify the expression patterns of waterlogging tolerance candidate genes under waterlogging stress, RT-qPCR was performed, and the quantitative results were consistent with the expression trends of RNA-seq gene expression data.

## 4 Discussion

Water is one of the key factors affecting plant growth and development, and leaves are the organs whose external morphology is most sensitive to water stress. Long-term exposure of plants to soil environments saturated with water or near saturation will cause leaf wilting, yellowing, and loss (Ashraf, 2012). To explore the effects of waterlogging stress on the growth and ornamental value of *Clematis*, four varieties in the flowering stage were subjected to waterlogging stress treatment. The results showed that the flowers of the plants were wilted and drooped after waterlogging stress, and *C. lanuginosa*, *C. tientaiensis*, and *‘*Viva Polonia’ all maintained the ability to produce seeds, but some seeds were green and sterile. Studies have shown that melatonin plays a role in reproductive protection during flowering, and its content is highest in young flower organs or during flower development (Wang et al., 2022d). In *Datura metel* L., the highest levels of melatonin were found in the least-developed buds, and the concentration of melatonin decreased as the buds matured. When exposed to low-temperature stress, the content of melatonin in young flowers increased significantly, indicating that melatonin was involved in the protection of new reproductive organs (Murch et al., 2009). After exogenous melatonin spraying, *C. tientaiensis* had strong waterlogging tolerance, was able to flower, and could maintain normal growth, while the seeds of *‘*Viva Polonia’ recovered the green color, indicating that melatonin may also play a role in protection of reproductive structures *of Clematis* under waterlogging stress and in maintaining normal reproductive growth.

Under normal conditions, the four types of *Clematis* showed differences in Pn and Gs, but no significant differences in Tr or Ci (**Figures 4B, D, F, H**). There were significant differences in leaf pigment content (**Supplementary Figure S1**), possibly due to differences in physiological characteristics among different species and varieties of *Clematis*. Waterlogging stress leads to hypoxia, stomatal conductance decrease, stomatal closure, leaf wilting, and decreased water evaporation. With the continuous waterlogging stress, the chlorophyll content decreases; leaves undergo premature aging, photosynthetic parameters (Pn, Gs, Ci, and Tr) are decreased, and the rate of photosynthesis decreases. Under waterlogging stress, the Chlb and total Chlorophyll content in the leaves of *C. lanuginosa* was significantly higher than in *C. tientaiensis*, the contents of Chla and Car were not significantly different (**Supplementary Figure S1**), and there were no significant differences in photosynthetic gas exchange parameters (**Figure 4B**), indicating that the photosynthetic system of *C. tientaiensis* may be more efficient than that of *C. lanuginosa*, or *Clematis* mainly absorbs and uses red light for photosynthesis. The specific differences still need to be further studied.

Melatonin can inhibit the degradation of chlorophyll under abiotic stress, improve the photosynthetic rate of leaves by enhancing the activity of Rubisco, and promote the accumulation of plant dry matter (Yang et al., 2018). Melatonin can decrease the expression of the key gene of chlorophyll degradation, *pheophorbide A oxygenase* (*PAO*), and increase the expression of the key photosynthetic gene RBCS, thereby improving the photosynthetic efficiency of plants under drought stress (Cherono et al., 2021). Exogenous foliar spraying of melatonin increased the chlorophyll content and the maximum mass seed yield (Fv/Fm) of photosystem II photochemistry of *Sorghum bicolor* seedlings, thereby increasing the rate of photosynthesis (Zhang et al., 2022). The net photosynthetic rate of waterlogged *Medicago sativa* pretreated with melatonin significantly increased (Zhang et al., 2019). Melatonin maintained the chlorophyll content in *Clematis* under waterlogging stress and improved the photosynthetic capacity. The Pn of the four types of *Clematis* under the T1 treatment had the least difference (**Figure 4B**). Under waterlogging stress, the Ci values of *‘*Sen-No-Kaze’ and *‘*Viva Polonia’ were significantly lower than those of *C. lanuginosa* and *C. tientaiensis*, and melatonin spraying narrowed the gap between the varieties (**Figure 4F**). Different *Clematis* showed variation in melatonin concentration. *C. tientaiensis* and *‘*Viva Polonia’ had relatively strong waterlogging tolerance, and the chlorophyll content and photosynthetic capacity were significantly improved under the T3 treatment, while the low melatonin concentration did not result in a significant effect. *C. lanuginosa* and *‘*Sen-No-Kaze’ were improved by the medium and low melatonin concentrations (**Figures 3**; **4**). The results indicated that suitable addition of melatonin could improve the photosynthetic efficiency of *Clematis* to provide the energy needed for growth under waterlogging stress.

When plants grow under normal conditions, ROS activity is maintained. Under waterlogging stress, the original dynamic balance of ROS in plants is broken, resulting in membrane lipid peroxidation, increased membrane permeability, and leakage of plant cellular contents. Eventually, root rot, leaf wilting, and other forms of plant damage occur (Pan et al., 2021; Peláez-Vico et al., 2023). Plants can clear ROS through both enzymatic and non-enzymatic systems. Enzyme systems include SOD, CAT, POD, APX, glutathione peroxidase (GPX), glutathione reductase (GR), dehydroascorbate reductase (DHAR), and monodehydroascorbate reductase (MDHAR). Non-enzymatic systems eliminate ROS through the circulation of Ascorbate (AsA) and Glutathione (GSH) (Hossain et al., 2009; Foyer and Noctor, 2011). SOD, APX, and CAT activities of *Musa acuminata*, *Citrus reticulata*, and *Chrysanthemum morifolium* in leaves under waterlogging stress were higher than those of controls (Teoh et al., 2022; Li et al., 2023). The APX, POD, CAT, and SOD activities of *Clematis* were higher than those of the control group under waterlogging stress consistent with results from other species.

Melatonin can regulate the activity of antioxidant enzymes, remove free radicals, improve the redox state of cells, reduce ROS and RNS levels, and stabilize biofilms (Li et al., 2015). In *Malus pumila*, external application of melatonin reduced the physiological damage to the plant by ROS, thereby improving its stain resistance (Zheng et al., 2017). Exogenous melatonin effectively increased the activities of SOD, POD, and APX in *Zea mays*, *Prunus persica*, and *Sorghum bicolor* under waterlogging stress, and improved plant tolerance (Gu et al., 2021; Ahmad et al., 2022). In this study, exogenous melatonin improved the activities of APX, POD, SOD, and CAT in *Clematis* under waterlogging stress. By increasing the ability to remove ROS, the level of H_2_O_2_ was reduced, and the value of REC was decreased (**Figure 2**), and reduced the effect of waterlogging stress on the cell membrane system and enzyme function, indicating that oxidative damage was alleviated. Melatonin can inhibit the accumulation of ROS in *Clematis* under waterlogging stress to a certain extent, reduce the degree of membrane lipid peroxidation, reduce the damage to cell membranes, and alleviate the damage from waterlogging.

In this study, *Clematis* varieties with different waterlogging stress tolerance had different responses in antioxidant enzyme activity. The APX and SOD activity of ‘Sen-No-Kaze’ increased most significantly after melatonin spraying (**Supplementary Figure S2**), and the POD activity of ‘Sen-No-Kaze’ was higher than that of the other three types under normal culture conditions, waterlogging stress, and low melatonin concentration. With the increase in the melatonin concentration, the POD enzyme activity of ‘Sen-No-Kaze’ in the T2 and T3 treatments increased less than those of *C. lanuginosa* and *C. tientaiensis*. The POD activity of *C. lanuginosa* increased most significantly after melatonin spraying (**Supplementary Figure S2B**). The CAT antioxidant oxidase activity levels of *C. lanuginosa* and *C. tientaiensis* were higher than those of the other two varieties under waterlogging stress and melatonin treatment (**Supplementary Figures S2C, D**). The antioxidant enzyme activity of *Clematis* with strong waterlogging tolerance increased significantly after the addition of melatonin, while the antioxidant enzyme activity of *Clematis* with poor waterlogging tolerance was inhibited under the high melatonin concentration compared to the low concentration, a result that may be related to differences in tolerance.

The variety and growth stages of plants may have different effects on the amount of melatonin, and a high concentration of melatonin may inhibit plant growth (Arnao and Hernández-Ruiz, 2018). The leaf pigment content, APX, POD, and other antioxidant enzyme activities of *C. lanuginosa* under the T3 treatment were higher than those under the T1 treatment, but the relative water content of T3 was lower than that of T1 (**Supplementary Figure S5A**). This difference resulted in relatively high leaf pigment content, stomatal conductance, net photosynthetic rate, and antioxidant enzyme activity. Leaf pigment content, photosynthetic reaction gas parameters, and antioxidant enzyme activity decreased with the increase of melatonin concentration in ‘Sen-No-Kaze’, *C. lanuginosa* under T3 displayed partially mature leaf tips with yellowing. The reason for this may be that the high melatonin concentration inhibited the growth and development of *Clematis*, and the plants did not respond to waterlogging stress or the low melatonin treatment as under the relatively high melatonin concentration. Moreover, the ‘Sen-No-Kaze’ variety was more sensitive to the high melatonin concentration, but the specific differences in response need to be further studied.

Non-enzymatic antioxidant protective substances include proline, soluble protein, and soluble sugar. As the main osmoregulatory substances of plants, they can synergistically improve the activity of related enzymes or act as the substrates of related enzymes while reducing the osmotic potential of cell cytoplasm, promoting the absorption of water from the outside of cells, and regulating cell osmotic pressure to reduce the damage from waterlogging stress. Thus, the survival ability of plants under waterlogging stress can be improved (Wang et al., 2019). In studies of kiwifruit, *Capsicum annuum*, and other plants, exogenous application of melatonin increased the content of osmoregulatory substances in plants under stress, thus alleviating the damage caused by stress and helping the plants survive (Moustafa-Farag et al., 2020; Huo et al., 2022; Khosravi et al., 2023). Waterlogging stress significantly increased the contents of proline, soluble protein, and soluble sugar in the four *Clematis* (**Figure 6**). The levels of osmoregulatory substances in *C. tientaiensis* were significantly higher than those in *C. lanuginosa*, and those in ‘Viva Polonia’ were significantly higher than those in ‘Sen-No-Kaze’ Among the four species, the proline content of *C. tientaiensis* maintained relatively high level after waterlogging stress and melatonin induction (**Supplementary Figure S3A**). The soluble sugar contents in *C. lanuginosa* and ‘Sen-No-Kaze’ were decreased by the increase of melatonin concentration (**Supplementary Figure S3B**), indicating that the proline and soluble protein of *Clematis* with strong waterlogging tolerance were most significantly responsive to waterlogging stress, and may also improve the efficiency of energy use. Melatonin can be used as an effective plant biostimulant to combat biological and abiotic stresses because it can promote the accumulation of soluble sugar and proline. In this study, after spraying melatonin, the proline content, soluble sugar, and soluble protein content of *Clematis* were enhanced, thus effectively maintaining cell homeostasis and improving the adaptability of *Clematis* to waterlogging stress.

Transcription factors (TFs) are key regulators of gene expression, and they play a crucial role in regulating plant responses to various abiotic stresses (He et al., 2023). *C. lanuginosa* and *C. tientaiensis* are the original breeding parents of *Clematis*. In this study, stress resistance genes in wild *Clematis* were further extracted by transcriptome analysis. Under waterlogging stress and melatonin treatment, 23 and 14 transcription factors were differentially expressed in *C. lanuginosa* and *C. tientaiensis*, respectively. The two differentially expressed genes were distributed in *MYB*, Zinc finger protein, *ERF*, *FAR1*, *bHLH*, *WRKY*, *LOB*, and *B3* gene families (**Figure 8**). These differentially expressed transcription factors may be the key to the regulation of waterlogging tolerance of *Clematis tiantianensis*.

The transcription factor LOB (Lateral organ boundaries domain) has been shown to respond to waterlogging stress. Expression levels of LOB domain (LBD) family genes in *Solanum* L. and *Arabidopsis thaliana* increased under waterlogging stress (Dawood et al., 2016; Voesenek et al., 2016). In this study, *LBD4* in *C. lanuginosa* and *C. tientaiensis* were significantly positively correlated with the antioxidant enzyme system and were significantly up-regulated under waterlogging stress as well as being induced by melatonin, indicating that *LBD4* played an important role in improving the antioxidant enzyme activity of *Clematis* under waterlogging stress.

The AP2/ERF (APETALA2/ethylene-responsive factor) transcription factor family is critical for plant abiotic stress response (Licausi et al., 2013; Wang et al., 2022b). The family contains four subfamilies of AP2, RAV, ERF, and DREB, among which *ABR1* was responsive to ABA and stress conditions including cold, high salt, and drought (Pandey et al., 2005). In Arabidopsis, *AtABR1* was shoot specifically induced upon submergence and hypoxia, and overexpressing plants showed morphological phenotypes like increased root hair length and number, and directly or interaction with ABF4 promoted starch degradation to resist drought stress (Bäumler et al., 2019; Zhang et al., 2023b). In this study, *ABR1* was significantly negatively correlated with the photosynthetic system in *C. lanuginosa* and *C. tientaiensis*, and its expression was up-regulated by waterlogging stress induction, and the expression was inhibited by melatonin. These results suggest that *ABR1* may play an important role in *Clematis* waterlogging stress, and that melatonin can regulate the expression of *ABR1* to improve the tolerance to waterlogging.

MYB (v-MYB avian myeloblastosis viral oncogene homolog) transcription factor is one of the largest transcription factors in plants, and it plays important roles in the plant cell cycle, environmental response, stress response, primary metabolism, and secondary metabolism (Jung et al., 2008; Dubos et al., 2010; An et al., 2020). Studies have shown that the MYB protein REVEILLE/LHY-CCA1-Like (RVE1/LCL1), which is related to the biological clock, binds to the Evening element (EE) in the DREB1 promoter region, and RVE1/RVE2 plays a negative regulatory role in plant cold tolerance. It can also mediate the expression of the downstream *DREB1* gene under cold stress (Kidokoro et al., 2021). In this study, *RVE1* was significantly negatively correlated with the photosynthetic system in *C. lanuginosa* and significantly positively correlated with the antioxidant system in *C. tientaiensis*, suggesting that *RVE1* in *Clematis* may negatively regulate waterlogging tolerance and mediate the expression of *DREB1b*. MOF1 is a transcriptional suppressor that can interact with three TOPLESS related proteins, namely, *OsTPR1*, *OsTPR2*, and *OsTPR3*, inhibit the expression of *DL* and *OsMADS6*, and thus inhibit flower organogenesis and spikelet meristem development in rice (Li et al., 2020; Ren et al., 2020). The expression of *MOF1* was up-regulated under waterlogging stress, and the appropriate concentration of melatonin inhibited the expression of *MOF1*, which restored the organ development ability of *Clematis*.

Transposase-derived transcription factors FRS-FRF family proteins play crucial roles in plant growth and development in processes, such as oxidation reactions, starch synthesis, flowering time, flower development, drought stress, low phosphorus response, and ABA response (Ma and Li, 2018). FRS-FRF family proteins are divided into six subgroups according to their protein structure. *FRS5* is in the V subfamily (Lin et al., 2007; Li et al., 2011; Tang et al., 2013; Aguilar-Martínez et al., 2015; Liu et al., 2017). Up-regulation of the FRS5 gene in *Triticum aestivum* increased its tolerance to nitrogen deficiency (Wang et al., 2021). In this study, waterlogging stress inhibited the expression of FRS5 (**Figure 8**), while melatonin induced up-regulation of *FRS5*, and this improved the waterlogging tolerance of *Clematis*.

The plant-specific transcription factor DOF (DNA binding with one finger) protein family is a classic plant-specific transcription factor family belonging to the single zinc finger structural protein superfamily. The proteins are involved in the regulation of different plant processes during growth and development (Gupta et al., 2015; Boccaccini et al., 2016). Overexpression of *MdDOF54* in apple *(Malus × domestica)* would result in a higher photosynthetic rate and stronger water transport capacity under long-term drought conditions than that of the wild type, which greatly increased the survival rate under short-term drought conditions (Chen et al., 2020). DOF proteins are also key regulatory centers of various plant hormone pathways, integrating abscisic acid, jasmonic acid, salicylic acid, and redox signaling (Wang et al., 2022c). In this study, the inhibition of *DOF36* expression by waterlogging stress was positively correlated with photosynthesis, while the significant upregulation of *DOF47* was negatively correlated with photosynthesis, suggesting that DOF36 may positively regulate photosynthesis, similar to *MdDOF54*, while *DOF47* negatively regulates photosynthesis.

WRKYs are the largest and most important family of transcription factors in plants and are primarily involved in responses to various stresses (Rushton et al., 2010; Wang et al., 2021). Recent studies have shown that members of this gene family are involved in hypoxic stress responses in many plant species, including *Oryza sativa* (Shiono et al., 2014), *Helianthus annuus* (Raineri et al., 2015), and *Diospyros kaki* (Zhu et al., 2019). WRKY33 in *Arabidopsis thaliana* roots was induced after hypoxia treatment, and *wrky33* mutants were sensitive to hypoxia stress treatment (Hwang et al., 2011). In this study, *WRKY4* in *C. lanuginosa* was significantly positively correlated with the photosynthetic system, while WRKY4 in *C. tientaiensis* was significantly negatively correlated with the antioxidant system, suggesting that *WRKY4* may play a positive and positive regulating role in the photosynthetic system and negatively regulating the antioxidant system, thereby improving the tolerance to waterlogging in *Clematis*.

In this study, waterlogging stress damaged the membrane system, the photosynthetic system, and the oxidation balance of *Clematis*, significantly reducing photosynthetic production. Exogenous melatonin effectively reduced lipid peroxidation of the membrane of flooded *Clematis*, maintained photosynthetic production capacity, improved antioxidant enzyme activity, and improved the adaptability of *Clematis* to waterlogging stress. From the perspective of economy and production, 50 μmol·L^-1^ can enhance the waterlogging tolerance of *Clematis* to a certain extent to meet the production demand, and nine transcription factors that can improve the waterlogging tolerance of *Clematis* were identified through transcriptome analysis. The findings thus provide an important basis and reference for the further application of melatonin in the cultivation and resistance breeding of *Clematis*. In future studies, it will be necessary to explore the regulatory role of these transcription factors on *Clematis* waterlogging tolerance to further improve our understanding of the underlying mechanisms.

## Data availability statement

The raw transcriptome data have been deposited at the NCBI Sequence Read Archive with accession number PRJNA1070854 (https://www.ncbi.nlm.nih.gov/bioproject/PRJNA1070854).

## Author contributions

KC: Conceptualization, Data curation, Formal analysis, Writing – original draft. QH: Funding acquisition, Project administration, Resources, Writing – review & editing. XM: Investigation, Methodology, Writing – original draft. XZ: Investigation, Methodology, Writing – original draft. RQ: Writing – review & editing. JZ: Writing – review & editing.

## Supplementary material

Supplementary Figure 1 Effects of waterlogging stress on the chlorophyll content of *Clematis*.

Supplementary Figure 2 Effects of waterlogging stress on antioxidant enzyme activity of *Clematis*.

Supplementary Figure 3 Effect of waterlogging stress on proline, soluble sugar, and soluble protein content of *Clematis*.

Supplementary Figure 4 GO annotation analysis of DEGs in *C. lanuginosa* and *Clematis*.

Supplementary Figure 5 Effect of waterlogging stress on relative water content of *Clematis*.
