# Supplementary figures and images for: The effect of exogenous melatonin on waterlogging stress in Clematis
Source: Front Plant Sci. 2024 Jun 18;15:1385165. doi: 10.3389/fpls.2024.1385165 (PMC11217522; doi:10.3389/fpls.2024.1385165)

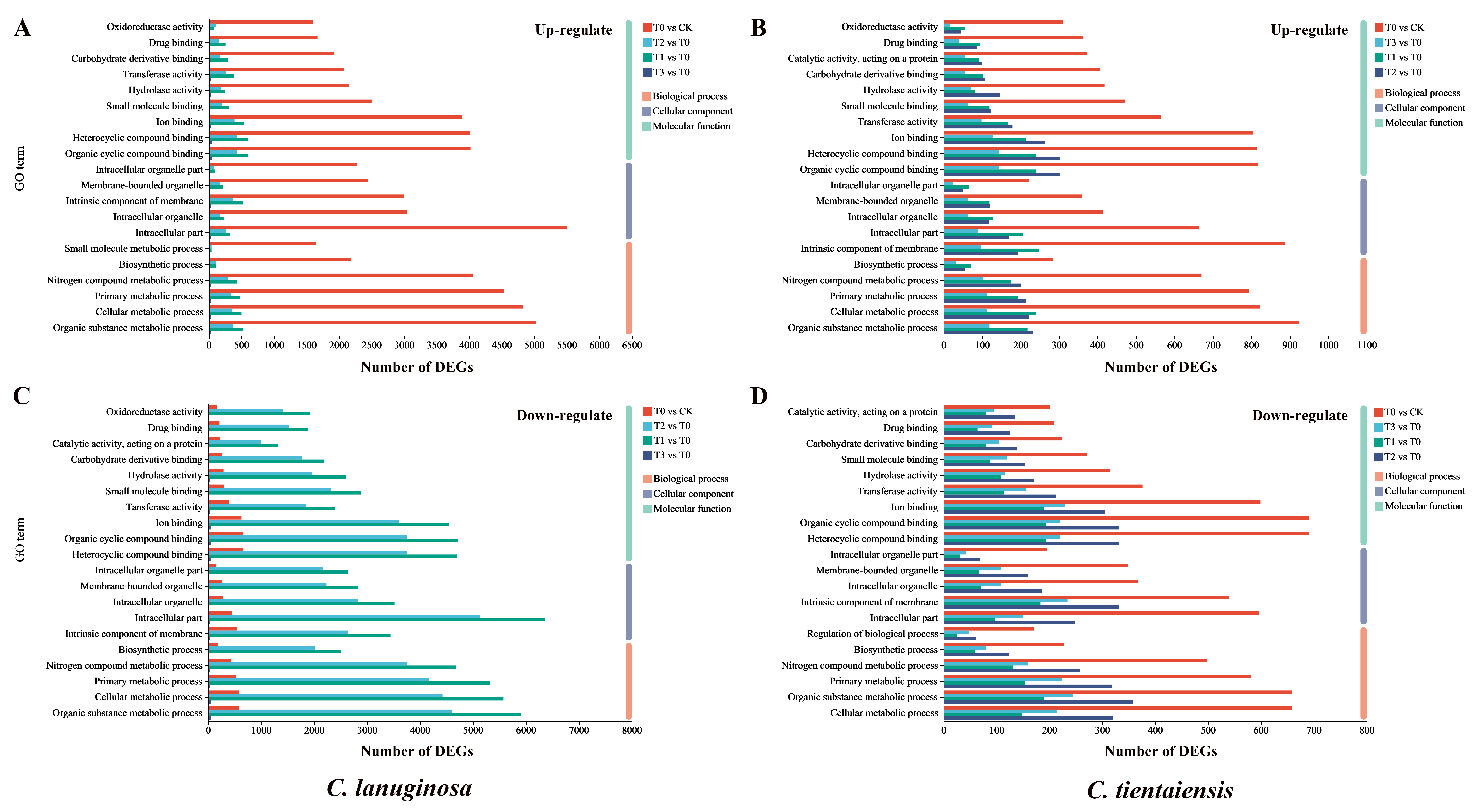

Supplement: Supplementary Figure 4 — GO annotation analysis of DEGs in C. lanuginosa and Clematis. [file Image_4.tif]

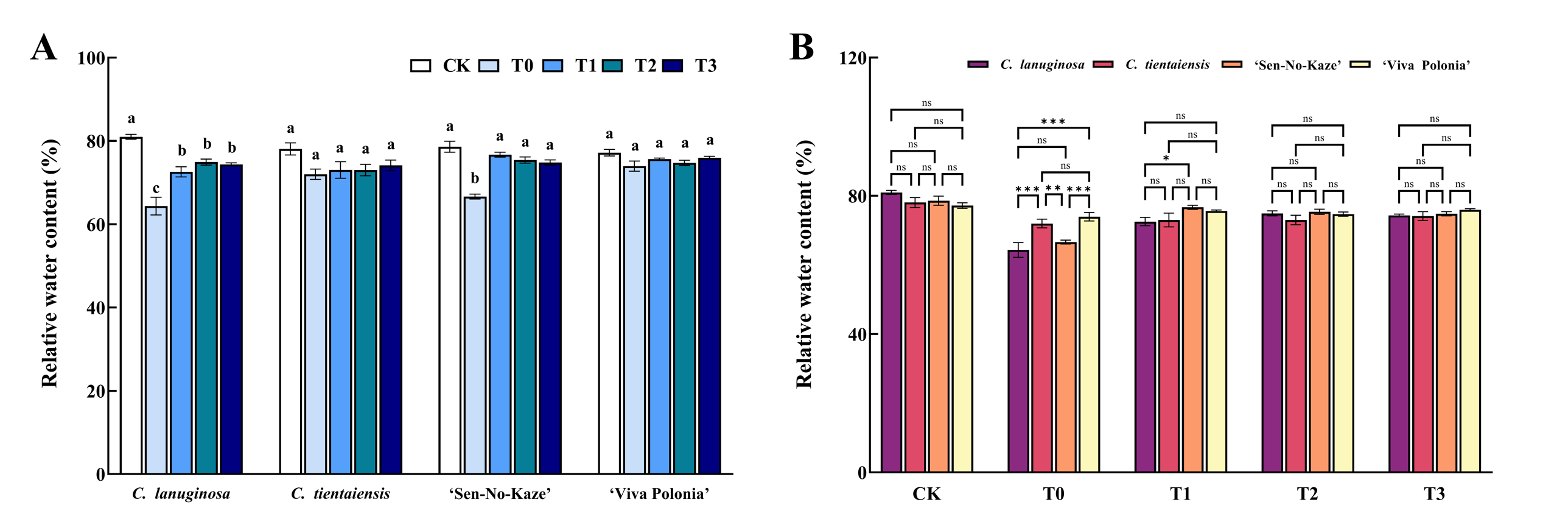

Supplement: Supplementary Figure 5 — Effect of waterlogging stress on relative water content of Clematis. [file Image_5.tif]
