# Supplementary material for: The effect of exogenous melatonin on waterlogging stress in Clematis
Source: Front Plant Sci. 2024 Jun 18;15:1385165. doi: 10.3389/fpls.2024.1385165 (PMC11217522; doi:10.3389/fpls.2024.1385165)
Supplement: Supplementary file 6 [file Table_1.docx]

**Table S1.** Summary of transcriptome sequencing data of *C. lanuginosa* and *C. tientaiensis*.

| Sample | Raw reads | Raw bases | Clean reads | Clean bases | Error rate(%) | Q20(%) | Q30(%) | GC content(%) |
| --- | --- | --- | --- | --- | --- | --- | --- | --- |
| A1_1 | 41337546 | 6241969446 | 40905030 | 6004209941 | 0.0251 | 97.46 | 95.41 | 45.59 |
| A1_2 | 47714882 | 7204947182 | 47235932 | 6896538787 | 0.0251 | 97.42 | 95.36 | 45.56 |
| A1_3 | 52145116 | 7873912516 | 51703586 | 7551614481 | 0.025 | 97.48 | 95.52 | 45.13 |
| A2_1 | 51579446 | 7788496346 | 51084678 | 7520176873 | 0.025 | 97.5 | 95.49 | 46.36 |
| A2_2 | 47842948 | 7224285148 | 47458542 | 6990864743 | 0.025 | 97.48 | 95.45 | 46.72 |
| A2_3 | 49554154 | 7482677254 | 49099888 | 7225618546 | 0.0252 | 97.4 | 95.31 | 47.69 |
| A3_1 | 46822310 | 7070168810 | 46440924 | 6877616668 | 0.025 | 97.47 | 95.41 | 45.59 |
| A3_2 | 48691046 | 7352347946 | 48241008 | 7089513978 | 0.0251 | 97.46 | 95.4 | 45.79 |
| A3_3 | 47996042 | 7247402342 | 47623410 | 6947720103 | 0.025 | 97.48 | 95.48 | 45.05 |
| A4_1 | 42227758 | 6376391458 | 41877910 | 6131121106 | 0.0252 | 97.42 | 95.32 | 45.41 |
| A4_2 | 43440588 | 6559528788 | 42994952 | 6263050102 | 0.0253 | 97.34 | 95.21 | 45.38 |
| A4_3 | 49579234 | 7486464334 | 49065146 | 7210619912 | 0.0252 | 97.43 | 95.3 | 45.63 |
| A5_1 | 45483768 | 6868048968 | 45020502 | 6604048911 | 0.0251 | 97.46 | 95.38 | 45.88 |
| A5_2 | 49992864 | 7548922464 | 49557746 | 7188320027 | 0.0251 | 97.45 | 95.43 | 46.93 |
| A5_3 | 42413312 | 6404410112 | 42035764 | 6122332946 | 0.025 | 97.48 | 95.5 | 46.21 |
| B1_1 | 45884134 | 6928504234 | 45447730 | 6661958168 | 0.0252 | 97.39 | 95.28 | 45.69 |
| B1_2 | 50495630 | 7624840130 | 50056166 | 7320354883 | 0.025 | 97.46 | 95.49 | 46.54 |
| B1_3 | 43761786 | 6608029686 | 43340448 | 6326774975 | 0.0251 | 97.43 | 95.36 | 46.18 |
| B2_1 | 44501688 | 6719754888 | 44092550 | 6462350346 | 0.0251 | 97.41 | 95.35 | 45.37 |
| B2_2 | 45186180 | 6823113180 | 44735134 | 6605885111 | 0.0251 | 97.44 | 95.34 | 45.55 |
| B2_3 | 51675412 | 7802987212 | 51051890 | 7499425766 | 0.025 | 97.5 | 95.47 | 44.74 |
| B3_1 | 47901474 | 7233122574 | 47352680 | 6938677461 | 0.0253 | 97.38 | 95.23 | 46.24 |
| B3_2 | 44147958 | 6666341658 | 43762010 | 6457373954 | 0.0251 | 97.45 | 95.38 | 45.21 |
| B3_3 | 48647992 | 7345846792 | 48142516 | 7029704183 | 0.0253 | 97.33 | 95.22 | 46.25 |
| B4_1 | 44086036 | 6656991436 | 43638536 | 6466679656 | 0.025 | 97.51 | 95.46 | 45.37 |
| B4_2 | 57545680 | 8689397680 | 57019124 | 8352153970 | 0.0249 | 97.51 | 95.53 | 45.56 |
| B4_3 | 50949658 | 7693398358 | 50507276 | 7231009936 | 0.0252 | 97.43 | 95.35 | 44.85 |
| B5_1 | 45163346 | 6819665246 | 44693094 | 6557150992 | 0.0252 | 97.4 | 95.25 | 45.46 |
| B5_2 | 47999226 | 7247883126 | 47578208 | 7001155916 | 0.0251 | 97.46 | 95.42 | 45.54 |
| B5_3 | 44284120 | 6686902120 | 43896004 | 6498354390 | 0.0252 | 97.41 | 95.3 | 45.6 |
