# Supplementary material for: The effect of exogenous melatonin on waterlogging stress in Clematis
Source: Front Plant Sci. 2024 Jun 18;15:1385165. doi: 10.3389/fpls.2024.1385165 (PMC11217522; doi:10.3389/fpls.2024.1385165)
Supplement: Supplementary file 7 [file Table_2.docx]

**Table S2.** Statistics of splicing results.

| Type | Unigene | Transcript |
| --- | --- | --- |
| Total number | 129833 | 199498 |
| Total base | 153185608 | 239096966 |
| Largest length (bp) | 19868 | 19868 |
| Smallest length (bp) | 201 | 201 |
| Average length (bp) | 1179.87 | 1198.49 |
| N50 length (bp) | 1718 | 1747 |
| E90N50 length (bp) | 2641 | 2194 |
| Fragment mapped percent(%) | 68.836 | 82.848 |
| GC percent (%) | 42.99 | 42.67 |
| TransRate score | 0.29923 | 0.34744 |
| BUSCO score | C:81.6%[S:77.6%;D:4.0%] | C:93.1%[S:49.4%;D:43.7%] |
