# Supplementary material for: The effect of exogenous melatonin on waterlogging stress in Clematis
Source: Front Plant Sci. 2024 Jun 18;15:1385165. doi: 10.3389/fpls.2024.1385165 (PMC11217522; doi:10.3389/fpls.2024.1385165)
Supplement: Supplementary file 8 [file Table_3.docx]

**Table S3.** Transcription factor gene family information

| Gene_id | PF ID | DNA domain | Description | Family | E-value | Score |
| --- | --- | --- | --- | --- | --- | --- |
| TRINITY_DN37514_c0_g1 | PF02701 | Zf-Dof | Dof domain, zinc finger | C2C2 | 2.90E-31 | 107.5 |
| TRINITY_DN42936_c0_g1 | PF02701 | Zf-Dof | Dof domain, zinc finger | C2C2 | 1.60E-27 | 95.6 |
| TRINITY_DN29894_c0_g1 | PF00010 | HLH | Helix-loop-helix DNA-binding domain | bHLH | 1.30E-10 | 41.3 |
| TRINITY_DN23024_c0_g1 | PF00249 | Myb_dna_bind | Myb-like DNA-binding domain | MYB_superfamily | 1.20E-20 | 73.6 |
| TRINITY_DN14333_c0_g1 | PF00249 | Myb_dna_bind | Myb-like DNA-binding domain | MYB_superfamily | 4.70E-30 | 103.7 |
| TRINITY_DN12800_c0_g1 | PF00249 | Myb_dna_bind | Myb-like DNA-binding domain | MYB_superfamily | 2.90E-07 | 30.7 |
| TRINITY_DN106_c0_g1 | PF00249 | Myb_dna_bind | Myb-like DNA-binding domain | MYB_superfamily | 5.50E-33 | 113.1 |
| TRINITY_DN5965_c0_g1 | PF00249 | Myb_dna_bind | Myb-like DNA-binding domain | MYB_superfamily | 4.20E-14 | 52.6 |
| TRINITY_DN16236_c0_g1 | PF00096 | zf-C2H2 | Zinc finger, C2H2 type | C2H2 | 3.00E-19 | 68.5 |
| TRINITY_DN52792_c0_g2 | PF00096 | zf-C2H2 | Zinc finger, C2H2 type | C2H2 | 2.60E-11 | 43.4 |
| TRINITY_DN53268_c0_g1 | PF00096 | zf-C2H2 | Zinc finger, C2H2 type | C2H2 | 5.30E-10 | 39.3 |
| TRINITY_DN64231_c0_g1 | PF03101 | FAR1 | FAR1 DNA-binding domain | FAR1 | 3.90E-17 | 62.8 |
| TRINITY_DN8016_c0_g1 | PF03101 | FAR1 | FAR1 DNA-binding domain | FAR1 | 0.00091 | 20 |
| TRINITY_DN8746_c0_g1 | PF03106 | WRKY | WRKY DNA -binding domain | WRKY | 3.40E-51 | 171.5 |
| TRINITY_DN40279_c0_g1 | PF00847 | AP2 | AP2 domain | AP2/ERF | 3.70E-13 | 49.7 |
| TRINITY_DN7576_c0_g1 | PF00847 | AP2 | AP2 domain | AP2/ERF | 4.80E-15 | 55.7 |
| TRINITY_DN89545_c0_g1 | PF00319 | SRF-TF | SRF-type transcription factor (DNA-binding and dimerisation domain) | MADS | 2.20E-21 | 75.2 |
| TRINITY_DN60693_c1_g1 | PF00170 | bZIP_1 | bZIP transcription factor | bZIP | 2.40E-08 | 34.1 |
| TRINITY_DN14497_c0_g1 | PF00170 | bZIP_1 | bZIP transcription factor | bZIP | 1.80E-07 | 31.3 |
| TRINITY_DN13315_c0_g1 | PF00170 | bZIP_1 | bZIP transcription factor | bZIP | 0.00037 | 20.7 |
| TRINITY_DN4041_c0_g1 | PF00447 | HSF_dna_bind | HSF-type DNA-binding | HSF | 2.60E-19 | 69.7 |
| TRINITY_DN1621_c0_g1 | PF03195 | DUF260 | Lateral organ boundaries (LOB) domain | LOB | 2.20E-10 | 41.1 |
| TRINITY_DN122_c3_g2 | PF02362 | B3 | B3 DNA binding domain | B3_superfamily | 1.50E-19 | 69.9 |
