# Supplementary material for: The effect of exogenous melatonin on waterlogging stress in Clematis
Source: Front Plant Sci. 2024 Jun 18;15:1385165. doi: 10.3389/fpls.2024.1385165 (PMC11217522; doi:10.3389/fpls.2024.1385165)
Supplement: Supplementary file 9 [file Table_4.docx]

**Table S4.** Transcription factor annotation information

| Gene ID | length | GO_id | GO_term | Go_description | KO_id | KO_name | EggNOG_id | EggNOG_Functional Categories | NR_hit-name | NR_description | Swiss-Prot_hit-name | Swiss-Prot_description | Pfam_id | Domain | Domain description |
| --- | --- | --- | --- | --- | --- | --- | --- | --- | --- | --- | --- | --- | --- | --- | --- |
| TRINITY_DN40279_c0_g1 | 926 | GO:0005634;;GO:0003700;;GO:0003677; | cellular_component;;molecular_function;;molecular_function;; | CC:nucleus;;MF:transcription factor activity, sequence-specific DNA binding;;MF:DNA binding;; | ------ | ------ | ENOG410YJG4 | K:Transcription) | KAF9591150.1 | hypothetical protein IFM89_002105 [Coptis chinensis]) | sp\|P93835\|DRE1B_ARATH;; | Dehydration-responsive element-binding protein 1B OS=Arabidopsis thaliana OX=3702 GN=DREB1B PE=1 SV=2;; | PF00847;;PF16014 | AP2;;SAP130_C | AP2 domain;;Histone deacetylase complex subunit SAP130 C-terminus |
| TRINITY_DN8016_c0_g1 | 1241 | GO:0006355;;GO:0005634;;GO:0016021;;GO:0008270;;GO:0003676; | biological_process;;cellular_component;;cellular_component;;molecular_function;;molecular_function;; | BP:regulation of transcription, DNA-templated;;CC:nucleus;;CC:integral component of membrane;;MF:zinc ion binding;;MF:nucleic acid binding;; | ------ | ------ | ENOG410ZJSX | S:Function unknown) | KAF5184748.1 | Far1-related sequence 5-like [Thalictrum thalictroides]) | sp\|Q9SZL8\|FRS5_ARATH;; | Protein FAR1-RELATED SEQUENCE 5 OS=Arabidopsis thaliana OX=3702 GN=FRS5 PE=1 SV=1;; | PF03101 | FAR1 | FAR1 DNA-binding domain |
| TRINITY_DN1621_c0_g1 | 2829 | ------ | ------ | ------ | ------ | ------ | ENOG410YHFG | S:Function unknown) | KAF5192590.1 | Lob domain-containing protein [Thalictrum thalictroides]) | sp\|Q9SHE9\|LBD4_ARATH;; | LOB domain-containing protein 4 OS=Arabidopsis thaliana OX=3702 GN=LBD4 PE=1 SV=1;; | PF03195 | LOB | Lateral organ boundaries (LOB) domain |
| TRINITY_DN106_c0_g1 | 1058 | GO:0003677; | molecular_function;; | MF:DNA binding;; | K09422 | MYBP | COG5147 | K:Transcription) | PIA37535.1 | hypothetical protein AQUCO_03000245v1 [Aquilegia coerulea]) | sp\|Q7XBH4\|MYB4_ORYSJ;; | Transcription factor MYB4 OS=Oryza sativa subsp. japonica OX=39947 GN=MYB4 PE=2 SV=2;; | PF00249;;PF13921;;PF15963 | Myb_DNA-binding;;Myb_DNA-bind_6;;Myb_DNA-bind_7 | Myb-like DNA-binding domain;;Myb-like DNA-binding domain;;Myb DNA-binding like |
| TRINITY_DN37514_c0_g1 | 900 | GO:0005634;;GO:0003700;;GO:0003677; | cellular_component;;molecular_function;;molecular_function;; | CC:nucleus;;MF:transcription factor activity, sequence-specific DNA binding;;MF:DNA binding;; | ------ | ------ | ENOG410YN5P | S:Function unknown) | PIA30654.1 | hypothetical protein AQUCO_05400034v1 [Aquilegia coerulea]) | sp\|Q84K52\|DOF47_ARATH;; | Dof zinc finger protein DOF4.7 OS=Arabidopsis thaliana OX=3702 GN=DOF4.7 PE=1 SV=1;; | PF02701 | zf-Dof | Dof domain, zinc finger |
| TRINITY_DN12800_c0_g1 | 1531 | GO:0005634;;GO:0003700;;GO:0003677; | cellular_component;;molecular_function;;molecular_function;; | CC:nucleus;;MF:transcription factor activity, sequence-specific DNA binding;;MF:DNA binding;; | ------ | ------ | ENOG410YDJI | K:Transcription) | PIA64022.1 | hypothetical protein AQUCO_00201368v1 [Aquilegia coerulea]) | sp\|A3AWH5\|MOF1_ORYSJ;; | Myb family transcription factor MOF1 OS=Oryza sativa subsp. japonica OX=39947 GN=MOF1 PE=1 SV=1;; | PF00249 | Myb_DNA-binding | Myb-like DNA-binding domain |
| TRINITY_DN42936_c0_g1 | 2954 | GO:0005634;;GO:0003700;;GO:0003677; | cellular_component;;molecular_function;;molecular_function;; | CC:nucleus;;MF:transcription factor activity, sequence-specific DNA binding;;MF:DNA binding;; | ------ | ------ | ENOG4111CPJ;;ENOG410ZMIT | K:Transcription);;S:Function unknown) | XP_028757594.1 | dof zinc finger protein DOF1.6-like [Prosopis alba]) | sp\|Q9M2U1\|DOF36_ARATH;; | Dof zinc finger protein DOF3.6 OS=Arabidopsis thaliana OX=3702 GN=DOF3.6 PE=1 SV=2;; | PF02701 | zf-Dof | Dof domain, zinc finger |
| TRINITY_DN8746_c0_g1 | 2764 | GO:0005634;;GO:0003700;;GO:0043565; | cellular_component;;molecular_function;;molecular_function;; | CC:nucleus;;MF:transcription factor activity, sequence-specific DNA binding;;MF:sequence-specific DNA binding;; | ------ | ------ | ENOG410YFS5 | K:Transcription) | PIA38758.1 | hypothetical protein AQUCO_02700161v1 [Aquilegia coerulea]) | sp\|Q9XI90\|WRKY4_ARATH;; | Probable WRKY transcription factor 4 OS=Arabidopsis thaliana OX=3702 GN=WRKY4 PE=1 SV=2;; | PF03106 | WRKY | WRKY DNA -binding domain |
| TRINITY_DN29894_c0_g1 | 1296 | GO:0006357;;GO:0005634;;GO:0003700;;GO:0046983;;GO:0003677; | biological_process;;cellular_component;;molecular_function;;molecular_function;;molecular_function;; | BP:regulation of transcription from RNA polymerase II promoter;;CC:nucleus;;MF:transcription factor activity, sequence-specific DNA binding;;MF:protein dimerization activity;;MF:DNA binding;; | ------ | ------ | ENOG41114KF;;ENOG41108WX | K:Transcription);; | KAF5196140.1 | Transcription factor bhlh [Thalictrum thalictroides]) | sp\|Q9FLI1\|BH036_ARATH;; | Transcription factor bHLH36 OS=Arabidopsis thaliana OX=3702 GN=BHLH36 PE=2 SV=1;; | PF00010 | HLH | Helix-loop-helix DNA-binding domain |
| TRINITY_DN5965_c0_g1 | 2029 | GO:0003677; | molecular_function;; | MF:DNA binding;; | ------ | ------ | ENOG410YHV7 | K:Transcription) | PIA63928.1 | hypothetical protein AQUCO_00201329v1 [Aquilegia coerulea]) | sp\|F4KGY6\|RVE1_ARATH;; | Protein REVEILLE 1 OS=Arabidopsis thaliana OX=3702 GN=RVE1 PE=1 SV=1;; | PF00249;;PF15963 | Myb_DNA-binding;;Myb_DNA-bind_7 | Myb-like DNA-binding domain;;Myb DNA-binding like |
| TRINITY_DN7576_c0_g1 | 3144 | GO:0009873 | biological_process;;cellular_component;;molecular_function;;molecular_function;; | BP:ethylene-activated signaling pathway;;CC:nucleus;;MF:transcription factor activity, sequence-specific DNA binding;;MF:DNA binding;; | ------ | ------ | ENOG411198J | K:Transcription) | PIA41179.1 | hypothetical protein AQUCO_02300168v1 [Aquilegia coerulea] | sp\|Q70II3\|EF110_ARATH | Ethylene-responsive transcription factor ERF110 OS=Arabidopsis thaliana OX=3702 GN=ERF110 PE=2 SV=2 |  |  |  |
